# Association between exercise variations and depressive symptoms among precarious employees in South Korea

**DOI:** 10.1038/s41598-021-95383-y

**Published:** 2021-08-05

**Authors:** Jae Won Oh, Jin Young Park, San Lee

**Affiliations:** 1 Department of Psychiatry, Yongin Severance Hospital, Yongin, South Korea; 2 Mind Health Clinic, Yongin Severance Hospital, Yongin, South Korea; 3 Department of Psychiatry and the Institute of Behavioral Science in Medicine, Yonsei University College of Medicine, Seoul, South Korea

**Keywords:** Psychology, Health care, Health occupations

## Abstract

Research regarding the association between depression and exercise has been limited regarding precariously employed individuals. The current study investigated the association between exercise variations and depressive symptoms among precarious employees in South Korea. Data from the 2014, 2016, and 2018 Korea National Health and Nutrition Examination Survey (KNHANES) were analyzed. In total, 13,080 participants aged ≥ 19 years responded to the survey. The Korean version of the PHQ-9 was utilized in addition to questions assessing regular exercise. Precariously employed men engaging in two or more variations of exercise each week were significantly less likely to report depressive symptoms (adjusted (OR): 0.78; 95% CI 0.62–0.97; p = 0.025), and the likelihood of depression was also lower for women who engaged in one or more forms of exercise (adjusted OR: 0.82; 95% CI 0.71–0.94; p = 0.006). These findings support the association between depression and exercise and suggest that greater variations in regular exercise are associated with a reduction in depression for men whereas any form of exercise reduces the risk of depression in women.

## Introduction

Employment stability has been a significant contributing factor in socioeconomic status, which directly influences one’s health status^1^. Precarious employment is considered as a determinant of health, as it not only influences the health and well-being of employees but also their families and associated communities^2^. The negative effects of precarious employment have been associated with a higher prevalence of mental health conditions including depression and anxiety^3, 4^, in addition to reduced health-related quality of life^5^. Such mental health consequences have become one of the greatest health problems leading to the global socioeconomic burden^6, 7^.

Depression may affect a person’s well-being in addition to impairing daily and professional functioning^8^. Furthermore, depression can not only directly result in considerable costs including healthcare services, but it can also have an indirect and substantial impact resulting in reduced productivity, work capacity, and even early retirement^9, 10^. Studies on depression among employees have shown that depression is a major cause of long-term sickness absence^11^. Thus, studies have highlighted the importance of health promotion including counseling services, stress management, and improvement in work environment settings to prevent depression among employees, although these have only displayed minor effects^12^. Furthermore, work-directed interventions including work adjustments and additional coaching provision have also demonstrated promising outcomes^13^.

Reduced physical activity is determined to be one of the main features of depression and is included in the diagnostic criteria of depression^14^. Previous research has suggested that exercise may play a role in the reduction of depression^15^; thus, numerous studies have focused on physical activity interventions to reduce depressive symptoms, at least within the clinical population^16, 17^. Supervised strength training have also demonstrated positive effects on depression in comparison to relaxation^18^, whereas aerobic exercise did not demonstrate any effects^19^. Likewise, recent research suggests that anaerobic exercise also helps to reduce depressive symptoms^20^.

Nonetheless, longitudinal studies have failed to identify the consistent effects of physical exercise on the psychological symptoms of the general population^17^. Moreover, whilst studies have assessed workplace exercise interventions to reduce the risk of depression among sedentary employees and improve work-related outcomes such as the increase in work attendance and reduction of job stress^21^, these were mostly focused on permanent employees, without the consideration of factors surrounding precarious employment^8^. As a result, many aspects of how physical exercise prevents depression remain unclear^22^; with almost no studies to show the impacts of exercise among precarious employees.

Beyond the existing bilateral relationship between exercise and depression, previous research has demonstrated the efficacy of various exercise combinations in greatly improving physical health as compared to engaging in no exercise or only a single type of exercise^23, 24^. Therefore, the following study will investigate whether a combination of different exercises will have subsequent effects on the levels of depressive symptoms among precariously employed individuals in the South Korean population.

In addition, sleep plays a vital role in maintaining homeostasis and also enhancing mental and physical well-being^25^. However, sleep disturbances are typical among most depressed individuals with studies demonstrating a strong bilateral association between sleep and depression^26^. Additionally, many precarious employees are at risk of such sleep disturbances due to their work hours and also insecurities^27, 28^. Henceforth, understanding the importance of sleep on one’s well-being, the association of sufficient sleep and depressive symptoms will also be evaluated for the current study.

## Results

### Descriptive statistics

Among the participants, 12.0% of men and 19.7% of women scored 5 or above on the PHQ-9 scale for depressive symptoms. Both men and women who engaged in more variations of exercise reported a reduced prevalence of depressive symptoms in comparison to those not engaging in regular exercise. Among men, those who reported excessive amounts of sleep demonstrated the greatest prevalence of depressive symptoms in comparison to insufficient and normal levels of sleep (20.4% vs. 12.0% vs. 11.1%, *p* = 0.026). However, those with insufficient sleep had highest depressive symptom scores, followed by excessive and then normal levels (20.5% vs. 19.5% vs. 16.9%, *p* = 0.005) for female employees. Overall, both men and women with depressive symptoms reported lower levels of educational attainment, lower household income, current smokers, more likely to be unmarried and be diagnosed with a metabolic syndrome. While there was no significant difference according to residential area among men, for women, those living in rural areas reported a greater prevalence of depressive symptoms (21.6% vs. 19.3%, *p* = 0.048). The details are provided in Table 1.

**Table 1:** Sociodemographic characteristics of study participants and depression (PHQ-9 ≥ 5).

	Men			Women		
	Non-depressive	Depressive	*p*-value	Non-depressive	Depressive	*p*-value
**Exercise variations**			**0.006**			** < 0.001**
None	1457 (86.1)	236 (13.9)		2548 (77.2)	754 (22.8)	
One	1125 (88.4)	148 (11.6)		1585 (81.6)	358 (18.4)	
Two or more	1644 (89.5)	192 (10.5)		1998 (83.6)	392 (16.4)	
**Sleep**			**0.026**			**0.005**
Insufficient (< 6 h/day)	3183 (88.0)	435 (12.0)		4593 (79.5)	1185 (20.5)	
Normal (< 9 h/day)	965 (88.9)	121 (11.1)		1398 (83.1)	285 (16.9)	
Excessive (≥ 9 h/day)	78 (79.6)	20 (20.4)		140 (80.5)	34 (19.5)	
**Age (years)**			** < 0.001**			** < 0.001**
20–29	526 (86.1)	85 (13.9)		552 (72.8)	206 (27.2)	
30–39	441 (83.8)	85 (16.2)		902 (79.8)	228 (20.2)	
40–49	561 (86.3)	89 (13.7)		1142 (84.8)	205 (15.2)	
50–59	727 (89.3)	87 (10.7)		1311 (83.2)	264 (16.8)	
60–69	1056 (91.2)	102 (8.8)		1195 (80.7)	286 (19.3)	
≥ 70	915 (87.7)	128 (12.3)		1029 (76.6)	315 (23.4)	
**Educational attainment**			**0.031**			** < 0.001**
Elementary school and below	785 (85.2)	136 (14.8)		1609 (75.6)	519 (24.4)	
Middle school	578 (88.8)	73 (11.2)		687 (80.0)	172 (20.0)	
High school	1578 (88.3)	210 (11.7)		2004 (81.5)	454 (18.5)	
University or above	1285 (89.1)	157 (10.9)		1831 (83.6)	359 (16.4)	
**Equalized household income**			** < 0.001**			** < 0.001**
Quartile 1 (low)	833 (81.0)	195 (19.0)		1168 (71.4)	468 (28.6)	
Quartile 2	1177 (88.2)	157 (11.8)		1616 (79.3)	421 (20.7)	
Quartile 3	1170 (90.1)	129 (9.9)		1743 (83.3)	349 (16.7)	
Quartile 4 (high)	1046 (91.7)	95 (8.3)		1604 (85.8)	266 (14.2)	
**Marital status**			** < 0.001**			** < 0.001**
Married	3147 (90.5)	332 (9.5)		4406 (83.4)	875 (16.6)	
Separated/divorced/widowed	282 (77.9)	80 (22.1)		1080 (72.6)	408 (27.4)	
Never married	797 (82.9)	164 (17.1)		645 (72.6)	221 (25.5)	
**Alcohol use status**			0.829			0.819
No	1353 (87.9)	187 (12.1)		3709 (80.4)	905 (19.6)	
Yes	2873 (88.1)	389 (11.9)		2422 (80.2)	599 (19.8)	
**Smoking status**			** < 0.001**			** < 0.001**
Non-smoker	2954 (90.2)	322 (9.8)		5954 (81.2)	1377 (18.8)	
Smoker	1272 (83.4)	254 (16.6)		177 (58.2)	127 (41.8)	
**MetS**			** < 0.001**			** < 0.001**
No	3984 (88.4)	521 (11.6)		5099 (81.1)	1186 (18.9)	
Yes	242 (81.5)	55 (18.5)		1032 (76.4)	318 (23.6)	
**Residential area**			0.953			**0.048**
Urban	3319 (88.0)	453 (12.0)		5031 (80.7)	1201 (19.3)	
Rural	907 (88.1)	123 (11.9)		1100 (78.4)	303 (21.6)	
**BMI**			** < 0.001**			**0.002**
Underweight	102 (76.1)	32 (23.9)		256 (73.6)	92 (26.4)	
Normal weight	2473 (88.1)	334 (11.9)		4002 (81.3)	922 (18.7)	
Overweight	1435 (88.7)	183 (11.3)		1555 (79.2)	408 (20.8)	
Obesity	216 (88.9)	27 (11.1)		318 (79.5)	82 (20.5)	
**Participants**	4226	576		6131	1504	

Categorical variables are presented as numbers and percentages. Bold values denote a p-value of <.05.

*PHQ-9* patient health questionnaire-9, *MetS* metabolic syndrome, *BMI* body mass index.

### Gender differences in the association between exercise and depressive symptoms

Multivariable logistic regression was conducted to evaluate the association of depressive symptoms with exercise variations, as demonstrated in Table 2. Precariously employed men engaging in two or more variations of exercise each week were 0.78 times less likely to report depressive symptoms (adjusted Odd Ratio (OR): 0.78; 95% CI 0.62–0.97; *p* = 0.025), thus representing the association between exercise and the reduction of depressive symptoms. However, women who engaged in even just one form of exercise reported a reduced likelihood of depression (adjusted OR: 0.82; 95% CI 0.71–0.94; *p* = 0.006), as well as those engaged in two or more variations of exercise (adjusted OR: 0.72; 95% CI 0.62–0.83; *p* < 0.001).

**Table 2:** Results of the multivariable logistic regression analysis for the association between exercise and depressive symptoms in precarious employees.

	Depressive symptoms (PHQ ≥ 5)							
	Men				Women			
	OR	95% CI		*p*-value	OR	95% CI		*p*-value
**Exercise variations**								
None	1.00				1.00			
One	0.89	0.71	1.12	0.313	**0.82**	**0.71**	**0.94**	**0.006**
Two or more	**0.78**	**0.62**	**0.97**	**0.025**	**0.72**	**0.62**	**0.83**	** < 0.001**
**Sleep**								
Insufficient (< 6 h/day)	1.01	0.81	1.26	0.963	**1.19**	**1.03**	**1.38**	**0.020**
Normal (< 9 h/day)	1.00				1.00			
Excessive (≥ 9 h/day)	1.69	0.98	2.93	0.060	0.98	0.65	1.47	0.929
**Age (years)**								
20–29	1.00				1.00			
30–39	**1.56**	**1.07**	**2.29**	**0.021**	**0.72**	**0.54**	**0.96**	**0.025**
40–49	1.28	0.85	1.91	0.236	**0.49**	**0.36**	**0.66**	** < 0.001**
50–59	0.96	0.62	1.49	0.852	**0.46**	**0.34**	**0.62**	** < 0.001**
60–69	0.71	0.45	1.12	0.136	**0.40**	**0.29**	**0.55**	** < 0.001**
≥ 70	0.80	0.50	1.28	0.355	**0.33**	**0.23**	**0.47**	** < 0.001**
**Educational attainment**								
Elementary school and below	1.00				1.00			
Middle school	0.77	0.56	1.06	0.103	0.86	0.70	1.07	0.167
High school	**0.74**	**0.56**	**0.98**	**0.036**	**0.71**	**0.58**	**0.87**	**0.001**
University or above	0.76	0.55	1.03	0.080	**0.63**	**0.50**	**0.79**	** < 0.001**
**Equalized household income**								
Quartile 1 (low)	1.00				1.00			
Quartile 2	**0.58**	**0.45**	**0.74**	** < 0.001**	**0.69**	**0.58**	**0.82**	** < 0.001**
Quartile 3	**0.47**	**0.36**	**0.62**	** < 0.001**	**0.57**	**0.48**	**0.69**	** < 0.001**
Quartile 4 (high)	**0.40**	**0.30**	**0.54**	** < 0.001**	**0.51**	**0.42**	**0.62**	** < 0.001**
**Marital status**								
Married	1.00				1.00			
Separated/divorced/widowed	**2.13**	**1.38**	**2.56**	** < 0.001**	**1.52**	**1.30**	**1.78**	** < 0.001**
Never married	**1.88**	**1.38**	**2.84**	** < 0.001**	1.09	0.84	1.42	0.523
**Alcohol use status**								
No	1.00				1.00			
Yes	1.01	0.83	1.23	0.954	1.01	0.89	1.14	0.939
**Smoking status**								
Non-smoker	1.00				1.00			
Smoker	**1.62**	**1.34**	**1.97**	** < 0.001**	**2.57**	**2.01**	**3.29**	** < 0.001**
**MetS**								
No	1.00				1.00			
Yes	**2.07**	**1.49**	**2.88**	** < 0.001**	**1.20**	**1.02**	**1.42**	**0.033**
**Residential area**								
Urban	1.00				1.00			
Rural	0.90	0.72	1.13	0.370	0.96	0.83	1.12	0.608
**BMI**								
Underweight	**1.90**	**1.23**	**2.92**	**0.004**	**1.36**	**1.05**	**1.76**	**0.022**
Normal weight	1.00				1.00			
Overweight	0.97	0.80	1.19	0.776	1.03	0.89	1.18	0.712
Obesity	0.78	0.51	1.20	0.263	0.88	0.67	1.15	0.356

*PHQ-9* patient health questionnaire-9, *MetS* metabolic syndrome, *BMI* body mass index, *OR* odds ratio, *CI* confidence interval. Bold values denote a p-value of <.05.

Both men and women who were current smokers, underweight or had metabolic syndromes had higher likelihood of reporting depressive symptoms. Notably, insufficient sleep was not a significant factor for men; however, women who reported insufficient levels of sleep were 1.18 times more likely to have depressive symptoms (adjusted OR: 1.19; 95% CI 1.03–1.38; *p* = 0.020). Regarding marital status, women who were either separated, divorced, or widowed had significantly higher likelihood of reporting depressive symptoms whereas there was a higher possibility of depressive symptoms among men who were never married or were either separated, divorced, or widowed.

On the contrary, increased household income and higher educational attainment (high school for men and high school and above for women) reported a reduced likelihood of depressive symptoms.

### Combined effects of exercise variations and covariates on depressive symptoms

A subgroup analysis of the combined effects of exercise variations and each of the covariates on participants’ depressive symptom levels was performed. Table 3 reports the levels of analysis among men and Table 4 reports the outcomes among women. The association between sleep and exercise variations differed between each gender in terms of depressive symptoms. Only men having insufficient levels of sleep and engaged in two or more variations of exercise reported they were 0.72 times more likely to experience depressive symptoms (adjusted OR: 0.72; 95% CI 0.57–0.91; *p* = 0.005). Other sleep durations and exercise did not have any association with depressive symptoms.

**Table 3:** Subgroup analysis of the association between exercise variations on depressive symptoms (PHQ-9 ≥ 5) stratified by sociodemographic variables in men.

Men	None	One				Two or more			
	OR	OR	95% CI		*p*-value	OR	95% CI		*p*-value
**Sleep**									
Insufficient (< 6 h/day)	1.00	0.81	0.63	1.04	0.103	**0.72**	**0.57**	**0.91**	**0.005**
Normal (< 9 h/day)	1.00	0.79	0.49	1.29	0.349	0.70	0.45	1.09	0.114
Excessive (≥ 9 h/day)	1.00	1.06	0.32	3.57	0.921	1.41	0.43	4.59	0.569
**Age (years)**									
20–29	1.00	1.03	0.54	2.02	0.935	0.64	0.36	1.13	0.123
30–39	1.00	0.97	0.51	1.82	0.917	1.19	0.70	2.02	0.517
40–49	1.00	0.75	0.42	1.33	0.326	1.09	0.65	1.83	0.736
50–59	1.00	**0.55**	**0.31**	**0.98**	**0.041**	0.64	0.37	1.09	0.098
60–69	1.00	0.84	0.51	1.38	0.492	**0.61**	**0.38**	**1.00**	**0.049**
≥ 70	1.00	0.86	0.56	1.33	0.500	**0.45**	**0.28**	**0.72**	**0.001**
**Educational attainment**									
Elementary school and below	1.00	0.83	0.54	1.28	0.407	0.67	0.42	1.06	0.084
Middle school	1.00	1.41	0.79	2.49	0.243	0.95	0.52	1.75	0.871
High school	1.00	0.81	0.56	1.19	0.287	0.82	0.59	1.14	0.238
University or above	1.00	**0.59**	**0.38**	**0.92**	**0.020**	**0.60**	**0.41**	**0.88**	**0.008**
**Equalized household income**									
Quartile 1 (low)	1.00	0.97	0.66	1.41	0.859	0.72	0.49	1.05	0.087
Quartile 2	1.00	0.65	0.42	1.01	0.057	0.87	0.59	1.26	0.455
Quartile 3	1.00	0.88	0.56	1.38	0.571	0.68	0.44	1.05	0.080
Quartile 4 (high)	1.00	0.74	0.42	1.30	0.293	0.78	0.48	1.27	0.317
**Marital status**									
Married	1.00	0.84	0.64	1.10	0.195	**0.60**	**0.45**	**0.79**	** < 0.001**
Separated/divorced/widowed	1.00	0.66	0.35	1.22	0.185	0.65	0.36	01.17	0.151
Never married	1.00	0.78	0.53	1.17	0.406	0.78	0.53	1.17	0.230
**Alcohol use status**									
No	1.00	**0.67**	**0.46**	**1.00**	**0.047**	**0.68**	**0.48**	**0.98**	**0.036**
Yes	1.00	0.89	0.68	1.16	0.386	**0.74**	**0.58**	**0.95**	**0.019**
**Smoking status**									
Non-smoker	1.00	0.93	0.70	1.23	0.601	**0.67**	**0.51**	**0.88**	**0.004**
Smoker	1.00	0.74	0.52	1.05	0.095	0.98	0.72	1.33	0.877
**MetS**									
No	1.00	0.80	0.64	1.01	0.065	**0.73**	**0.59**	**0.90**	**0.003**
Yes	1.00	0.97	0.48	1.98	0.937	0.80	0.39	1.61	0.523
**Residential area**									
Urban	1.00	**0.69**	**0.54**	**0.89**	**0.004**	**0.64**	**0.51**	**0.81**	** < 0.001**
Rural	1.00	1.31	0.84	2.03	0.230	1.06	0.66	1.71	0.802
**BMI**									
Underweight	1.00	0.89	0.30	2.69	0.838	1.26	0.52	3.06	0.612
Normal weight	1.00	0.87	0.65	1.16	0.341	**0.75**	**0.58**	**0.98**	**0.038**
Overweight	1.00	0.79	0.54	1.16	0.236	**0.63**	**0.44**	**0.91**	**0.013**
Obesity	1.00	0.39	0.12	1.26	0.116	0.60	0.24	1.46	0.257

*PHQ-9* patient health questionnaire-9, *MetS* metabolic syndrome, *BMI* body mass index, *OR* odds ratio, *CI* confidence interval. Bold values denote a p-value of <.05.

**Table 4:** Subgroup analysis of the association between exercise variations on depressive symptoms (PHQ-9 ≥ 5) stratified by sociodemographic variables in women.

Women	None	One				Two or more			
	OR	OR	95% CI		*p*-value	OR	95% CI		*p*-value
**Sleep**									
Insufficient (< 6 h/day)	1.00	**0.77**	**0.66**	**0.90**	**0.001**	**0.65**	**0.56**	**0.76**	** < .001**
Normal (< 9 h/day)	1.00	0.86	0.63	1.18	0.350	0.82	0.61	1.11	0.196
Excessive (≥ 9 h/day)	1.00	**0.30**	**0.11**	**0.86**	**0.025**	**0.19**	**0.06**	**0.59**	**0.004**
**Age (years)**									
20–29	1.00	1.22	0.80	1.87	0.359	1.09	0.80	1.87	0.650
30–39	1.00	0.97	0.67	1.40	0.852	0.82	0.58	1.15	0.247
40–49	1.00	**0.68**	**0.47**	**0.99**	**0.044**	**0.51**	**0.36**	**0.73**	** < 0.001**
50–59	1.00	0.72	0.52	1.01	0.056	**0.68**	**0.49**	**0.92**	**0.014**
60–69	1.00	0.75	0.55	1.03	0.072	**0.56**	**0.41**	**0.78**	** < 0.001**
≥ 70	1.00	**0.59**	**0.43**	**0.82**	**0.001**	**0.56**	**0.39**	**0.81**	**0.002**
**Educational attainment**									
Elementary school and below	1.00	**0.78**	**0.61**	**1.00**	**0.049**	0.79	0.61	1.03	0.082
Middle school	1.00	0.70	0.47	1.06	0.092	**0.39**	**0.25**	**0.60**	** < .001**
High school	1.00	0.88	0.68	1.13	0.312	0.81	0.64	1.03	0.091
University or above	1.00	**0.72**	**0.54**	**0.97**	**0.029**	**0.68**	**0.53**	**0.89**	**0.004**
**Equalized household income**									
Quartile 1 (low)	1.00	**0.70**	**0.54**	**0.91**	**0.007**	**0.67**	**0.51**	**0.89**	**0.005**
Quartile 2	1.00	0.80	0.60	1.05	0.100	**0.78**	**0.60**	**1.00**	**0.050**
Quartile 3	1.00	**0.74**	**0.56**	**0.99**	**0.045**	**0.71**	**0.54**	**0.93**	**0.012**
Quartile 4 (high)	1.00	1.04	0.76	1.43	0.803	0.77	0.56	1.05	0.094
**Marital status**									
Married	1.00	**0.76**	**0.63**	**0.90**	**0.002**	**0.58**	**0.48**	**0.69**	** < 0.001**
Separated/divorced/widowed	1.00	**0.70**	**0.53**	**0.94**	**0.017**	**0.75**	**0.56**	**0.99**	**0.043**
Never married	1.00	1.03	0.69	1.55	0.880	0.97	0.67	1.38	0.849
**Alcohol use status**									
No	1.00	**0.68**	**0.57**	**0.81**	** < 0.001**	**0.60**	**0.50**	**0.72**	** < 0.001**
Yes	1.00	0.92	0.74	1.15	0.469	**0.77**	**0.62**	**0.94**	**0.012**
**Smoking status**									
Non-smoker	1.00	**0.79**	**0.69**	**0.92**	**0.002**	**0.65**	**0.57**	**0.75**	** < 0.001**
Smoker	1.00	0.58	0.31	1.10	0.097	1.09	0.64	1.86	0.740
**MetS**									
No	1.00	**0.78**	**0.67**	**0.92**	**0.003**	**0.73**	**0.63**	**0.84**	** < 0.001**
Yes	1.00	0.75	0.55	1.01	0.059	**0.47**	**0.33**	**0.67**	**0.001**
**Residential area**									
Urban	1.00	**0.70**	**0.59**	**0.82**	** < 0.001**	**0.63**	**0.54**	**0.73**	** < 0.001**
Rural	1.00	1.12	0.82	1.53	0.474	0.91	0.64	1.28	0.579
**BMI**									
Underweight	1.00	0.85	0.47	1.51	0.572	**0.55**	**0.31**	**0.99**	**0.047**
Normal weight	1.00	**0.81**	**0.68**	**0.96**	**0.018**	**0.65**	**0.55**	**0.77**	** < 0.001**
Overweight	1.00	**0.61**	**0.46**	**0.81**	** < 0.001**	**0.60**	**0.46**	**0.79**	** < 0.001**
Obesity	1.00	1.09	0.58	2.07	0.789	**1.89**	**1.08**	**3.31**	**0.026**

*PHQ-9* patient health questionnaire-9, *MetS* metabolic syndrome, *BMI* body mass index, *OR* odds ratio, *CI* confidence interval. Bold values denote a p-value of <.05.

However, both insufficient and excessive sleep was associated with likelihood of reduction in depressive symptoms for women when engaged in any form of exercise. Those who engaged in a single variation of exercise per week and reported having insufficient levels of sleep had a 0.77 times likelihood of reporting depressive symptoms (adjusted OR: 0.77; 95% CI 0.66–0.90; *p* = 0.001) and a 0.30 times likelihood of depressive symptoms for those having excessive amounts of sleep (adjusted OR: 0.30; 95% CI 0.11–0.86; *p* = 0.025). Interestingly, there was even a greater reduction in the likelihood for women engaged in two or more variations of exercise. Women with insufficient sleep reported a 0.65 times likelihood of reporting depressive symptoms (adjusted OR: 0.65; 95% CI 0.56–0.76; *p* =  < 0.001), whilst those who with excessive amounts of sleep had a likelihood of 0.19 for reporting depressive symptoms (adjusted OR: 0.19; 95% CI 0.06–0.59; *p* = 0.004). This demonstrates the importance of exercise and reduction of depressive symptoms regardless of the sleep levels of the individual, particularly for precariously employed women.

The combined effects of low educational attainment, lower income, non-alcohol drinker, non-smoker, living in urban areas, and exercise variations (both one and two or more variations) demonstrated a linear association with depressive symptoms among women. However, for men, household income had no association whereas the association of reduced depressive symptoms and exercise was presented for those with greater than university degree attainment. Regarding metabolic syndrome, the distinct gender effects have been identified. Men with metabolic syndromes did not demonstrate any effects of exercise on depression yet women who exercised on two or more variations had a significant reduction in the likelihood of having depressive symptoms (adjusted OR: 0.47; 95% CI 0.33–0.67; *p* = 0.001). Detailed subgroup analyses of male participants are presented in Table 3 and analyses of female participants are demonstrated in Table 4, illustrating the gender effects in the association between exercise variations and depressive symptoms.

## Discussion

Employment status and stability have been considered to be a significant indicator of one’s health and socioeconomic status^1^, where precarious employment is associated with an insecure form of employment, lower-income, and poorer working conditions^29^. This potentially leads to a higher prevalence of mental health conditions and reduced quality of life^3–5, 30^. Nonetheless, research on methods to support and promote the well-being of the precariously employed population have been limited. Henceforth the current research specifically examined the levels of depression and effects of exercise among precariously employed individuals within the South Korean population to address these risks.

The association between combinations of weekly exercise and the onset of depressive symptoms within the precariously employed population in South Korea was explored using 2014, 2016 and 2018 editions of the KNHANES. The current research aimed to identify associations between depressive symptoms and exercise variations including walking, strength exercise, and aerobic exercise. Both men and women reported a reduction in depressive symptoms when undertaking variations of physical exercise each week. The current findings were consistent with existing studies on physical activity being efficacious in reducing depressive symptoms^15^. In this study, there was a reduction in depressive symptoms among employees engaging in regular form physical activity.

However, this study was novel in its approach to investigate the association of weekly exercise combination on individuals’ depressive symptoms. Significant effects of exercise variations were detected within precarious employees. Upon close subgroup analysis, precariously employed men reported a reduction in depressive symptoms when engaging in two or more different variations of exercise whereas female employees who engaged in any single form of exercise or more per week displayed reduced depressive symptoms. Thus for men, exercise diversity and combination are vital, in comparison to women whose depressive symptoms were reduced even though they engaged in only one form of exercise.

In addition to regular exercise, reduction in sleep duration and quality have often been reported due to the shift in employment conditions towards requiring greater hours of work as well as night activity^28, 31^. This has significantly affected employees’ sleep patterns and increased fatigue, tiredness, and daytime sleepiness^31^. Existing research has established an association between sleep and depression, considering its complex and bidirectional nature and placing great emphasis on sleep duration and also the quality of sleep^32, 33^. Thus, in addition to the effects of exercise variation, the current study also investigated the impact of sleep on depressive symptoms as those in precarious employments are most at risk of lacking sleep and having poor sleep quality^27^. Upon conducting subgroup analyses, results showed that sleep did not affect the level of depressive symptoms among men. However, women with insufficient sleep (less than 6 h per day) reported an increased likelihood of depressive symptoms. This demonstrates the potential gender affect on the association between exercise and sleep of precarious employees, with women being more vulnerable to risks associated with insufficient sleep.

Additionally, while sleep did not have a direct association with depression among men, insufficient sleep coupled with two or more types of exercise was associated with rather reduced depressive symptoms. In contrast, for women with insufficient sleep, the subgroup analysis showed a reduction in depressive symptoms when this was coupled with both one type of exercise and two or more combinations, again demonstrating the importance of any form or combination of exercise in women. Furthermore, women engaging in two or more types of activity coupled with excessive sleep (9 h and more per day) also demonstrated a reduction in depressive symptoms. This highlights the importance of participating in exercise combination whereby, taking part in such exercise variations could potentially supplement the lack of sleep for men and women and reduce depressive symptoms.

In line with existing studies, consistent trends of reduction in depressive symptoms were associated with an increase in household income, whereas there was an increase in depressive symptoms among both men and women who were smokers, non-married (including divorced, separated, and widowed individuals), and underweight^1, 34, 35^. This study also identified the effects of exercise among those with metabolic syndromes. Whilst exercise was not an influential factor for men, women who participated in two or more diverse form of exercise activity had demonstrated a reduced likelihood of depressive symptoms.

The current study’s strengths include the following. Firstly, the study assessed the effects of exercise variation on the precariously employed population within the South Korean population. It was the first of the kind to investigate the combined effects of exercise rather than observing the effects of individual exercise types. Furthermore, prior studies have examined the association of depression and exercise among regular, permanent employees^8^ yet, specific attention to precarious employees have been absent in research. Consequently, this study has focused on the precariously employed population who have a higher risk of having problems related to mental and physical well-being^36, 37^. Hence, the current research has identified the direction that further research should take in order to support the well-being of these vulnerable employees. Moreover, the study used a nationwide population survey conducted over a 3 year period; thus, a large population was recruited and the sample may be representative of the South Korean population and the findings may be generalized^38^.

The study also has its limitations. Due to research being observational and correlational, it is unable to identify whether exercise variations reduced the likelihood of depressive symptoms or those with lower depressive scores were more likely to exercise. While it was imperative for this study, as the first original research, to identify this association, future research investigating causation among these variables should be conducted within the context of employment in South Korea. Furthermore, as the current study only involved precarious employees, future research comparing the association between exercise and depression for both precarious and permanent employees would provide additional information in managing the well-being of employees subject to their needs. Additionally, considering the innovative nature of the current research, grouping the physical activity responses into a new variable of exercise may have its limitations in assessing the combinations of exercise. More categories besides strength, aerobic, and walking exercises should be included to identify more specific features for the association between exercise and depression.

Overall, the current study demonstrated new and insightful findings regarding the relationships among exercise variations, sleep, and depressive symptoms in precarious employees. These associations were also moderated by gender, and the study also identified potential methods to reduce depression among precariously employed individuals. Based on the findings, precariously employed men may benefit in terms of mental well-being if they engage in various combinations of weekly physical exercises, whereas women can benefit from engaging in even just one type of exercise regularly. Further research should be conducted to determine the causality of the association on a longitudinal scale as well as identify specific types of physical activity that can significantly reduce the risks of depressive symptoms among precarious employees who may be at risk for depression.

## Methods

### Participants

Responses from the 6th and 7th Korea National Health and Nutrition Examination Survey (KNHANES), conducted by the Korea Disease Control and Prevention Agency were assessed. The survey was conducted amongst the non-institutionalized South Korean civilians throughout 192 regions to monitor the trends in health risk factors alongside the prevalence of major chronic disease and evaluate the health and nutritional status. In total, 10,000 individuals aged ≥ 1 year are selected for the survey each year and divided into three groups according to the stages of life: children (aged 1–11 years), adolescents (aged 12–18 years), and adults (aged 19 years and over). The database is publicly available on the KNHANES website (http://knhanes.cdc.go.kr).

**Figure 1:**
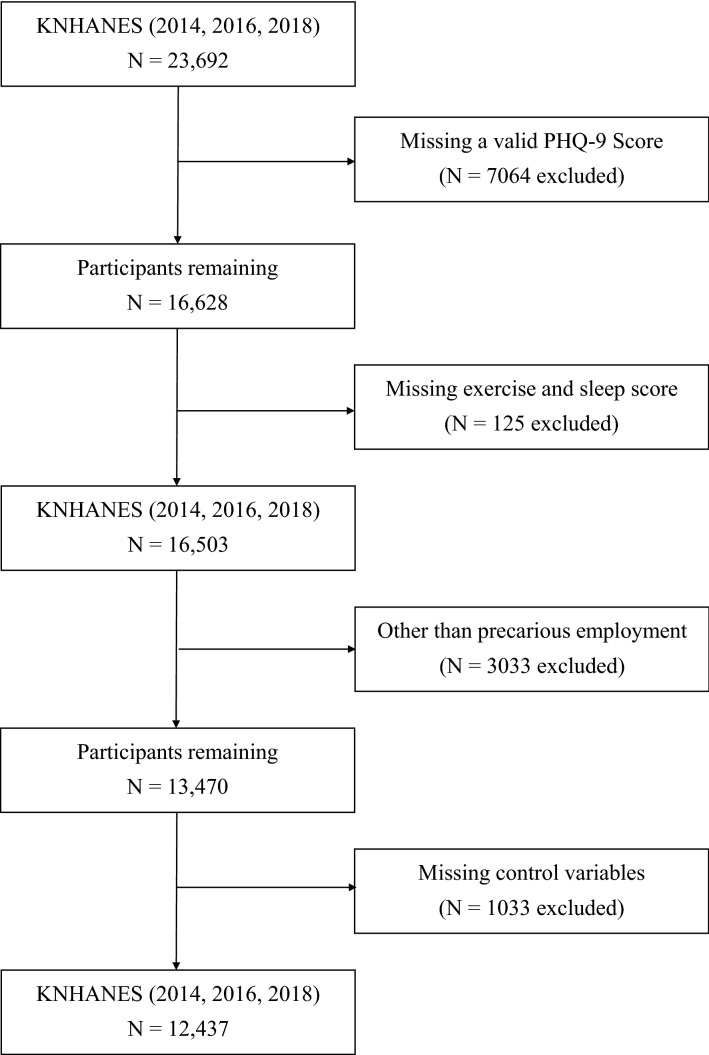
Flow diagram of the study participants.

The patient health questionnaire (PHQ-9) is administered biannually; hence, the current study used data from the 2014, 2016 and 2018 KNHANES. A total of 23,692 individuals responded, those without a valid PHQ-9 score (*n* = 7064), those not in precarious employment (*n* = 3033), without a valid exercise score (*n* = 71), missing sleep score (*n* = 54) and those missing covariate values (*n* = 1033) were excluded. Overall, there were 12,437 participants aged ≥ 19 years who were eligible for data analysis (Fig. 1). The Institutional Review Board of Yongin Severance Hospital waived the requirements for approval and consent because the analyses of the present study were based on de-identified, publicly available secondary data.

### Measures

#### Patient health questionnaire (PHQ-9)

The patient health questionnaire (PHQ-9) is a 9-item questionnaire that assesses the severity of depression with a total score ranging from 0 to 27. Each of the nine questions is scored between 0 (not at all) to 3 (nearly every day) and has been considered to be a reliable and valid measure to screen depression severity. Regarding the assessment of severity, PHQ-9 is comprised of five categories and cut-off scores of 0–4 indicates no depressive symptoms, 5–9 indicates mild depressive symptoms, 10–14 indicates moderate depressive symptoms, 15–19 moderate-severe depressive symptoms, and 20–27 indicates severe depressive symptoms^39^. A validation study of the Korean version of PHQ-9 which reported a cut-off score of 5 for screening depressive symptoms demonstrated 81.8% of sensitivity and 89.9% of specificity^40^. Henceforth, in the current study utilizing the Korean version of PHQ-9, the cut-off score of 5 was also applied to identify the minimal level of depressive symptoms within the participants.

Depression, according to DSM-5 criteria, is clinically met when five of the nine psychiatric symptoms are exhibited by the individuals during a 2-week period with at least one symptom being depressed mood or loss of interest^41^. In contrast subsyndromal depressive symptoms may not meet the criteria of a clinical disorder yet are associated with increased risk for the development of major depression or impairment^42^. Henceforth there are differences in the nomenclature of depressive symptoms and depression. For this study, depressive symptoms of precarious employees were assessed.

#### Exercise and sleep

Participants were assessed regarding their weekly exercise levels. Three different categories of exercises were evaluated which included aerobic exercise, strength exercise, and walking. Each participant was asked to answer a self-report measure, which included the number of days per week that they exercised for more than 10 min per session according to the type of exercise (strength, aerobic, and walking exercise). Strength measures included questions regarding whether the participants have done any form of exercise involving the muscles, including push-ups, sit-ups, weightlifting, pull-ups, etc. Any participants who have performed these exercises more than once per week were considered as undertaking regular physical activity whereas those who performed once or less were considered as non-exercising participants. Similar methods of assessment on exercise were conducted to assess the type of exercise that were associated with depression with a PHQ-9 cut-off score set at 10, demonstrating a significant association^22^. After assessing the exercise frequency and categorizing these into binary groups, the participants were then regrouped into those engaging in no form of exercise, one form of exercise, or two or more variations of exercise.

Sleep duration was self-reported, and participants were asked “how many hours of sleep do you usually get in a day on average?” From the responses, the durations were categorized as < 6 h/day, 6 to < 9 h/day, and ≥ 9 h/day. The questions and categories were in line with existing research association sleep duration and physical health within the South Korean adult population^43^.

#### Covariates

Demographic details (age and residential area), socio-economic status (education level, household income and marital status), and additional health-related variables (body mass index, smoking status, alcohol consumption and metabolic syndromes were assessed as covariates for the current study. The metabolic syndrome diagnosis was based on the recommendations of the presence of 3 or more of the following 5 abnormalities, (i) central obesity (waist circumference > 90 cm for men and > 80 cm for women); (ii) hypertension (blood pressure ≥ 140/90 mmHg or consuming antihypertensive drug treatment); (iii) hyperglycemia (fasting glucose level of serum ≥ 100 mg/dL or use of antidiabetic medication); (iv) high triglyceride (TG) levels (TG ≥ 150 mg/dL or drug treatment for dyslipidemia); or (v) low high-density lipoprotein cholesterol (HDL-C) levels (< 40 mg/dL among men and < 50 mg/dL among women).

### Statistical analyses

General characteristics of the participants were assessed using Chi-square tests. Multivariable logistic regressions examined the relationship between exercise variation and depressive symptoms. Subgroup analyses were also conducted to investigate the combined effects of exercise variation and the covariates on depressive symptoms. SAS software (version 9.4; SAS Institute, Cary, North Carolina, USA) was used for the analyses with a p-value of < 0.05.

## Data Availability

This study analysed data from the 2014, 2016 and 2018 KNHAES. All the KNHANES data are available to the public and can be downloaded from the KNHANES official website (http://knhanes.cdc.go.kr/).
